# Cross-sectional study of expression of divalent metal transporter-1, transferrin, and hepcidin in blood of smelters who are occupationally exposed to manganese

**DOI:** 10.7717/peerj.2413

**Published:** 2016-09-01

**Authors:** Qiyuan Fan, Yan Zhou, Changyin Yu, Jian Chen, Xiujuan Shi, Yanshu Zhang, Wei Zheng

**Affiliations:** 1 Department of Preventive Medicine, Zunyi Medical College, Zunyi, Guizhou, China; 2 Guizhou Institute of Occupational Safety and Health, Zunyi, Guizhou, China; 3 Department of Occupational Medicine, North China University of Science and Technology, Tangshan, Hebei, China; 4 School of Health Sciences, Purdue University, West Lafayette, IN, United States

**Keywords:** Manganese, Divalent metal transporter-1, Iron, Hepcidin, Occupational exposure, Human, Transferrin, Smelter

## Abstract

**Background:**

Manganese (Mn) is widely used in industries including the manufacture of Mn-iron (Fe) alloy. Occupational Mn overexposure causes manganism. Mn is known to affect Fe metabolism; this study was designed to test the hypothesis that workers exposed to Mn may have an altered expression of mRNAs encoding proteins in Fe metabolism.

**Methods:**

Workers occupationally exposed to Mn (*n* = 71) from a Mn–Fe alloy factory and control workers without Mn-exposure (*n* = 48) from a pig-iron plant from Zunyi, China, were recruited for this study. Blood samples were collected into Trizol-containing tubes. Total RNA was isolated, purified, and subjected to real-time RT-PCR analysis. Metal concentrations were quantified by atomic absorption spectrophotometry.

**Results:**

Working environment and genetic background of both groups were similar except for marked differences in airborne Mn concentrations (0.18 mg/m^3^ in Mn–Fe alloy factory vs. 0.0022 mg/m^3^ in pig-Fe plant), and in blood Mn levels (34.3 µg/L vs. 10.4 µg/L). Mn exposure caused a significant decrease in the expression of divalent metal transporter-1 (DMT1), transferrin (Tf) and hepcidin by 58.2%, 68.5% and 61.5%, respectively, as compared to controls, while the expression of transferrin receptor (TfR) was unaltered. Linear regression analysis revealed that expressions of DMT1, Tf and hepcidin were inversely correlated with the accumulative Mn exposure; the correlation coefficients (*r*) are −0.47, −0.54, and −0.49, respectively (*p* < 0.01).

**Conclusion:**

The data suggest that occupational Mn exposure causes decreased expressions of DMT1, Tf and hepcidin in blood cells; the finding will help understand the mechanism underlying Mn exposure-associated alteration in Fe homeostasis among workers.

## Introduction

Occupational exposure to high concentrations of Mn often occurs in Mn miners and smelters. Significant exposure also occurs in factories making Mn-iron (Fe) steel alloys or in welding practice (O’Neal & Zheng, 2015). Environmental exposure to Mn may be related to the use of the anti-knock gasoline additive methylcyclopentadienyl manganese tricarbonyl (MMT), Mn-containing pesticides and the clinical imaging contract agent mangafodipir trisodium (MnDPDP) (Aschner et al., 2007; O’Neal & Zheng, 2015).

Long-term Mn exposure causes manganism, characterized by progressive neurological disorders, with symptoms such as mask-like face, limb rigidity, mild tremors, gait disturbance and slurred speech, similar to Parkinson’s disease (Huang et al., 1993; Pal, Samii & Calne, 1999). While much effort has been devoted to characterization of the disease and understanding of the causation, the mechanism by which Mn causes neurodegenerative damage remains unknown.

Occupational exposure to Mn among smelting workers and welders has been associated with altered metal concentrations in whole blood, erythrocytes, plasma and saliva. For example, the concentrations of copper (Cu) in blood and saliva are significantly increased in smelters (Jiang et al., 2007) and welders (Wang, Du & Zheng, 2008), respectively, in comparison to control workers. Mn exposure also alters Fe concentrations in body fluids; however, the effect appears to vary significantly between different occupations. Among career welders, the Fe levels in serum are significantly increased (Li et al., 2004), whereas the smelters show a greatly reduced Fe concentrations in plasma as well as in erythrocytes (Cowan et al., 2009a; Cowan et al., 2009b). Animal studies report a reduced Fe level in blood but an increased Fe concentration in the CSF (Zheng et al., 1999; Li et al., 2006). An increased Fe level among welders is believed to be due to co-exposure to airborne Fe present in the welding fume. In contrast, a decreased Fe blood level in Mn-exposed animals is thought to be due to Mn interaction with Fe metabolism. The mimicry between Mn and Fe in their coordination chemistry allows Mn to compete with Fe in the fourth, labile Fe binding site in the active center of iron regulatory proteins (IRPs) and enhance the binding of IRPs to mRNAs containing iron responsive element (IRE) stem-loop structure, resulting in alteration of cellular Fe uptake (Li, Zhao & Zheng, 2005; Zheng, Ren & Graziano, 1998; Zheng & Zhao, 2001). Noticeably, the expression of Fe uptake-related proteins, transferrin receptor (TfR) and divalent metal transporter-1 (DMT1), is up-regulated in the choroidal epithelial cells that form the blood-cerebrospinal fluid barrier (Li, Zhao & Zheng, 2005; Wang, Li & Zheng, 2006)

DMT1 (also called Nramp2, DCT1 or Slc11a2) is an integral membrane phosphoglyco-protein and functions to transport a number of divalent metals (Fe^2+^, Mn^2+^, Co^2+^, Cu^2+^, Cd^2+^, Ni^2+^, Pb^2+^, and Zn^2+^) (Forbes & Gros, 2003). DMT1 proteins are mainly present in the plasma membrane of epithelial cells, such as the duodenum, kidney and choroid plexus, where it is essential for the absorption of divalent metals (Canonne-Hergaux et al., 2000; Canonne-Hergaux & Gros, 2002; Erikson et al., 2004; Wang, Li & Zheng, 2006; Wang, Du & Zheng, 2008). Transferrin (Tf) is mainly synthesized in the liver and subsequently released to the blood (Morgan, 1983). Other tissues, such as brain, testes and mammary glands, can also synthesize Tf. Tf functions as an Fe carrier in the plasma and interstitial fluids of the body; it accepts Fe released from cells and transports to the targeted cells where the Fe is taken up by TfR. Hepcidin is an endogenous antimicrobial peptide initially isolated from human blood and urine. Hepcidin has been shown to possess the ability to inhibit a cellular Fe efflux protein, ferroportin (i.e., MTP1) and to increases the cellular storage of Fe (Ganz, 2003). The response of these Fe regulatory proteins to variations of systemic Fe status has been established in literature (Adamsky et al., 2004; Gehrke et al., 2003; Theurl et al., 2008); however their expression at the translational level in the human body as affected by Mn exposure has never been investigated.

Given the evident Mn exposure-associated alteration in Fe homeostasis among smelting workers in Zunyi, China (Cowan et al., 2009a; Cowan et al., 2009b), we postulated that exposure to Mn in smelter workers may alter the expression of mRNAs encoding TfR, DMT1, Tf and/or hepcidin. Since the alteration of gene expression is likely a chronic effect, we further postulated that cumulative Mn exposure may be more relevant to the altered gene expression than the *ad hoc* blood concentrations of Mn and Fe. Thus, the purposes of this study were to investigate (1) whether the mRNA levels of selected Fe transport proteins were altered among Mn-exposed smelting workers in comparison to a control population, (2) whether the altered gene expression correlated to Mn/Fe concentrations in blood, and (3) whether the levels of Fe transport proteins changed as the function of cumulative Mn exposure. The findings from this study will help address the mechanism underlying chronic Mn exposure-associated alteration in Fe homeostasis among workers.

## Materials and Methods

### Study population

This cross-sectional human study was approved by the Office of Clinical Investigation at the ZMC and the Institutional Review Board for human study at Purdue University. Subjects were recruited from a Mn–Fe ferroalloy smelting factory for the Mn-exposed workers or from an Fe only smelting factory as the control subjects; both factories are located in Zunyi city of Guizhou province in China. Workers in both smelting factories usually operated the smelting process within 2–5 m of the smelting furnaces. The workers worked 7–8 h per day with the average employment history of 5.2 years. Both groups were also matched for socioeconomic status (salary, education, etc.) and background environmental factors (place of residence, etc.). Subjects in these two groups at the time of interview had no reported exposure to other toxic substances, radiation therapy or substance abuse. Subjects who had taken medications which could interfere with Fe metabolism, such as vitamin D, aspirin or herbal supplements, were excluded from the study.

### Determination of airborne Mn levels in work sites

Airborne Mn level were evaluated by individual sampler with the SKC pumps (model 224-44XR, calibrated at 2 L/min), tygon tubing and a sampling cassette with a close-face MCE filter (37 mm, pore size: 0.8 µm) within the worker’s breathing zone. A time-weighted average concentration was calculated following each 8 h work shift. Mn and Fe were determined by atomic absorption spectrophotometry (AAS) as briefly described below and in our published papers (Cowan et al., 2009a; Cowan et al., 2009b).

### Physical examination and blood sample collection

Clinical examination took place in the Zunyi Medical College, Zunyi, China. The written informed consent forms were obtained from all subjects prior to interview and physical examination. The study protocol received official approvals from the Office of Clinical Investigation at the ZMC and the human study Institutional Review Board at Purdue University. Participants agreed to use their blood samples for this biological monitoring research. The participants were asked to fast overnight prior to the study. Blood samples were collected in a heparinized tube and stood at room temperature for 30 min. After centrifuging at 600 g/min for 5 min, the supernatant and pellet were collected as the plasma fraction and the erythrocytes fraction, respectively for Mn and Fe analysis (Cowan et al., 2009a; Cowan et al., 2009b). For gene expression analysis, blood samples (0.5 ml of venous blood) were collected into autoclaved tubes (pre-loaded with 0.7 ml of Trizol). All samples were stored at −20 °C prior to laboratory analyses.

### Determination of Mn and Fe concentrations in air or blood samples

For analysis of Mn in air samples, the sampled filters were completely digested with 5 mL of HClO_4_–HNO_3_mixture (1:9 vol/vol) at 200 °C until dry, and the residues were dissolved in 10 mL of 1% HNO_3_ and diluted for AAS analysis.

For analysis of Mn and Fe in plasma, an aliquot (0.5 mL) of plasma was mixed with 5 mL of digestion solution (HClO_4_:HNO_3_; 3:7), stood at room temperature for 4–6 hr, and then heated until dry. The dried samples were then re-dissolved in 5 mL of 1% HNO_3_. For analysis of metals in erythrocytes, approximately 1.5 g of the pellet blood cells was mixed with saline to a volume of 4 mL. An aliquot (2 mL) of this fraction was mixed with 10 mL of the digestion solution and heated until dry; 5 mL of 1% HNO_3_ was added to dissolve the residue. The solution was diluted appropriately prior to atomic absorption spectrophotometry (AAS).

Mn and Fe concentrations were determined by AA240FS Varian AAS (Australia Pty Ltd.) using a China National Standard Operation Protocol (GB/T16018-1995) for occupational safety surveillance. The detection limits of graphite furnace AAS for Mn and Fe were 0.1 ng/mL and 0.09 ng/mL, respectively.

### Real-time RT-PCR analysis

The total RNA was extracted with TRIzol (Invitrogen, Carlsbad, CA), according to the Manufacturer’s recommendations, followed by purification process (Qiagen, Valencia, CA). The purified RNA was conveyed to cDNA with the High Capacity cDNA Reverse Transcription Kit (Applied Biosystems, Foster City, CA). The forward and reverse primer sequences for selected genes were designed with the ABI Primer Express software (Foster City, CA) and listed in Table 1. Power SYBR Green Master Mix (Applied Biosystems, Cheshire, UK) was used for real-time PCR analysis. The relative differences in expression between groups were expressed using cycle time (Ct) values as follows: the Ct values of the interested genes were first normalized with *β*-actin of the same sample, and then the relative differences between control and treatment groups were calculated and expressed as relative increases, setting control as 100%.

**Table 1:** Forward and reverse primer sequences for selected genes.

Gene product	Forward primer	Reverse primer
DMT1 (NM_000617)	CTCCACCATGACAGGAACT	AACAAGCAGAGTGGGGATGA
Tf (NM_001063)	GAAGCCTGCACTTTCCGTAG	AGACAGACGTGGTTAGCACA
TfR (NM_003234)	AGATTCCTGGTTCGGGTGTT	TGAAGAGACTCACTGCTGCA
Hepcidin (DQ496109)	CAAGACAACACTGCTGCACT	GAAGGACACACTTTGCACGT

**Notes.**

The forward and reverse primer sequences for selected genes were designed with the ABI Primer Express software (Foster City, CA).

### Data calculation and statistical analysis

Records of interviews and other reports were reviewed and abstracted for demographic data. All data are expressed as the mean ± SD unless otherwise stated. The cumulative Mn exposure for each worker was calculated by multiplying airborne Mn concentrations with years of employment, assuming 8 h per working day and 5 days per week. Linear regression models and Spearman correlation coefficients were used to evaluate the associations between mRNA expressions and Mn/Fe concentrations in plasma and erythrocytes, and between mRNA expressions and the accumulative Mn exposure, following the data transformation to logarithm. This transformation is valid with regards to the symmetric distribution and the linearity of variables after the logarithm transformation. Differences between two means were considered significant when *p* value was less than 0.05. Data were analyzed using SPSS13.0 software.

### Materials

Chemicals were obtained from the following sources: AAS standards of all metals from Alfa Products (Danvers, MA, USA). All reagents were of analytical grade, HPLC grade or the highest available pharmaceutical grade.

## Results

### Demographic and Mn exposure in the study population

The information on the study population, age and gender differences, Mn exposure and accumulated Mn exposures are summarized in Table 2. The subjects in Mn-exposed group were about 3 years younger than those of controls (*p* < 0.05). The ratio of male vs. female subjects in controls (5:1) is higher than that in the Mn-exposed group (3.7:1); however, this difference is not statistically significant. Daily monitoring of air samples using the stationary monitors indicated the mean airborne Mn concentrations of 0.002 mg/m^3^ and 0.18 mg/m^3^ in control and Mn-exposed workers, respectively. While the airborne Mn level was closed to the maximum allowable concentration (MAC) of 0.2 Mn mg/m^3^ in the work place by the standards of Chinese Ministry of Public Health (TJ36-79) and American Conference of Governmental Industrial Hygienists, the mean accumulative Mn exposure was about 214 fold higher in Mn-exposed workers than in the control group (Table 2).

**Table 2:** Summary of demographic data among Mn-exposed and control workers.

	Control	Mn-exposed
Number of subjects	48	71
Age (years) (95% CI)	37.2 ± 8.2 (34.5–39.8)	34 ± 5.3 (32.8–35.3)^*^
Gender (male/female)	40/8	56/15
Airborne Mn (mg/M^3^) (95% CI)	0.002 ± 0.002 (0.002–0.003)	0.18 ± 0.14 (0.13–0.24)^**^
Cumulative Mn exposure (g) (95% CI)	0.030 ± 0.015 (0.026–0.034)	6.42 ± 5.3 (4.40–8.44)^**^

**Notes.**

Data represent mean ± S.D.

* *p* < 0.05.

** *p* < 0.01 compared with the control group.

### Mn and Fe concentrations in the plasma and erythrocytes

Mn concentrations in plasma and erythrocytes were about 3.3 and 2.6 fold higher, respectively, in Mn-exposed workers than in controls (*p* < 0.05; Table 3). The Fe concentrations in Mn-exposed workers, on the other hand, were significantly decreased by 33% and 36% in plasma and erythrocytes, respectively, as compared to controls (Table 3). The results indicate that Mn exposure increased Mn concentrations while it decreased Fe concentrations in the blood compartment; the observation is consistent with our previous reports (Cowan et al., 2009a; Cowan et al., 2009b).

**Table 3:** Concentrations of Mn and Fe in plasma and erythrocyte.

	Mn		Fe	
	Plasma (mg/L)	Erythrocyte (ng/g)	Plasma (mg/L)	Erythrocyte (mg/g)
Control (95% CI)	10.4 ± 7.63 (8.02–12.8)	5.30 ± 3.50 (4.15–6.37)	0.94 ± 0.46 (0.81–1.09)	1.22 ± 0.51 (1.06–1.38)
Mn-exposure (95% CI)	34.3 ± 20.7^*^ (25.3–43.2)	13.8 ± 10.2^*^ (7.48–20.0)	0.63 ± 0.27^*^ (0.56–0.70)	0.78 ± 0.13^*^ (0.75–0.81)

**Notes.**

Data represent mean ± S.D.

* *p* < 0.05 compared with the control group.

### Expression of DMT1, Tf, TfR and hepcidin in blood cells

Among the genes involved in Fe metabolism, Tf molecules bind Fe and transport Fe in extracellular space; DMT1 and TfR function to take Fe into the cells; and hepcidin can increase intracellular Fe storage. Data by real-time RT-PCR showed that the relative transcript levels of DMT1,Tf and hepcidin in Mn-exposed workers were significantly decreased by 58%, 68% and 61%, respectively, as compared to controls (Table 4). However, the expression of TfR was not altered.

**Table 4:** Relative mRNA expressions of DMT1, Tf, TfR and hepcidin in blood cells.

	*N*	Control	*N*	Mn-exposed	% decreased
DMT1 (95% CI)	44	343.4 ± 314.1 (244.8–442.0)	61	143.6 ± 213.2^*^ (90.1–197.1)	58.2
Tf (95% CI)	39	477.3 ± 354.1 (366.2–588.4)	57	150.5 ± 222.0^*^ (92.9–208.1)	68.5
TfR (95% CI)	40	272.0 ± 167.3 (219.5–324.5)	58	257.0 ± 212.5 (202.3–311.7)	5.5
Hepcidin (95% CI)	36	555.1 ± 449.0 (414.2–696.0)	46	213.9 ± 318.8^*^ (121.8–306.0)	61.5

**Notes.**

For RT-PCR analyses, the DMT1, Tf and TfR gene expressions were determined first, followed by hepcidin. Due to the consumption of mRNA samples in earlier runs, for some subjects, there were not enough mRNA samples left for hepcidin assay. Thus, the total numbers for hepcidin were less than those for other three genes. Data represent mean ± S.D.

* *p* < 0.01 compared with the control group.

### Changes of mRNA levels of DMT1, Tf and hepcidin as the function of blood Mn and Fe levels

**Figure 1:**
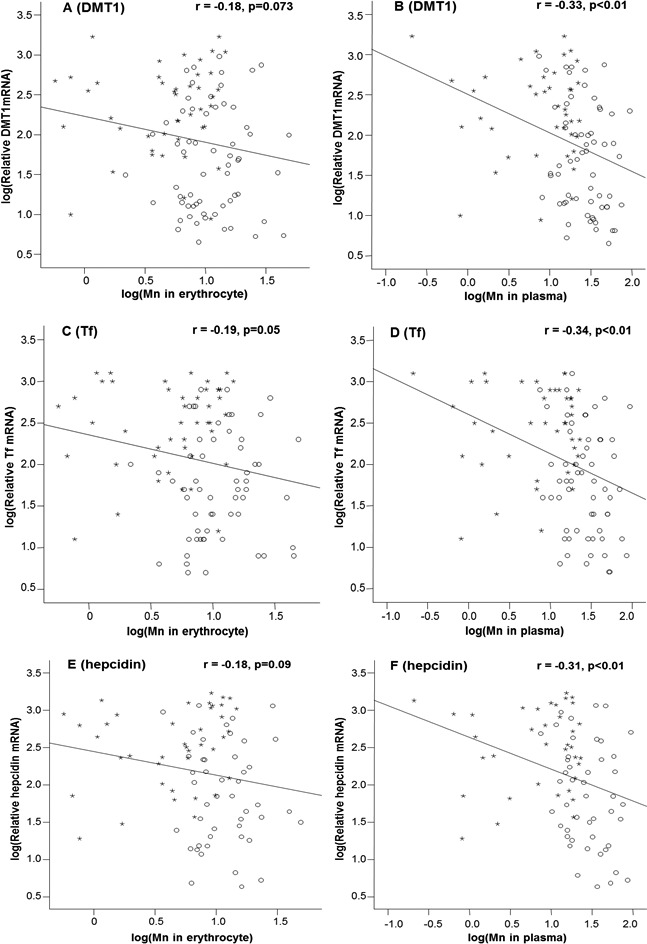
Correlations between DMT1, Tf and hepcidin expressions and Mn concentrations in the erythrocyte and in the plasma. Stars represent the control group and circles represent the Mn-exposure group. The DMT1 levels were inversely correlated with Mn concentrations in erythrocyte (A) and plasma (B), with the *r* values of −0.18 (*p* = 0.073) and −0.33 (*p* < 0.01), respectively. The Tf levels were inversely correlated with Mn concentrations in erythrocyte (C) and plasma (D) with the *r* values of −0.19 (*p* = 0.05) and −0.34 (*p* < 0.01), respectively. The hepcidin levels were also inversely correlated with Mn concentrations in erythrocyte (E) and plasma (F) with the *r* values of −0.18 (*p* = 0.09) and −0.31(*p* < 0.01), respectively. The correlations of DMT1, Tf and hepcidin with plasma Mn concentrations are apparently better than those with erythrocytic Mn concentrations.

By linear regression analysis, the levels of mRNAs encoding DMT1, Tf and hepcidin appeared to show the tendency to decline as the Mn concentrations in erythrocytes increased (Figs. 1A, 1C and 1E); however, the correlations between mRNA levels and erythrocytic Mn levels approached to, but did not achieve, the statistical significance, except for Tf mRNA (*p* = 0.05; Fig. 1C). For plasma samples, the mRNA expression levels of all 3 Fe regulatory transport proteins were inversely correlated with the Mn-concentrations in plasma (*p* < 0.01; Figs. 1B, 1D and 1F).

The changes of DMT1, Tf and hepcidin mRNA expressions as the function of Fe concentrations in erythrocytes and plasma were also analyzed; the results are summarized in Table 5. There was a general positive correlation between mRNA expressions and Fe concentrations in plasma and erythrocytes; however, none of these correlations reached the statistical significance (Table 5).

**Table 5:** Correlations between gene expressions and Fe concentration in blood.

	*n*	Erythrocyte Fe		Plasma Fe	
		*r*	*p*-value	*r*	*p*-value
DMT1	105	0.14	0.194	0.18	0.077
Tf	96	0.17	0.101	0.20	0.053
Hepcidin	82	0.22	0.060	0.14	0.190

**Notes.**

Data were analyzed by linear regression.

### Changes of biological parameters as the function of cumulative Mn exposure

**Figure 2:**
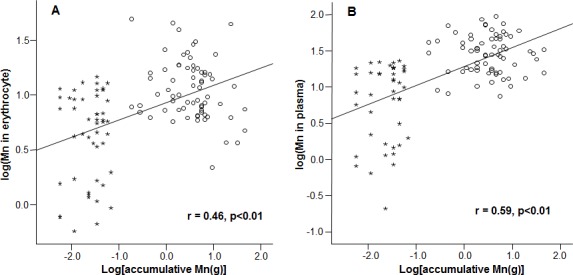
Correlations between accumulative Mn-exposure and Mn concentrations in erythrocyte and plasma. Stars represent the control group and circles represent the Mn-exposure group. Accumulative Mn-exposure was positively correlated with both Mn-concentration in the erythrocytes (A) and in plasma (B) with the *r* values of 0.46 (*p* < 0.01) and 0.59 (*p* < 0.01), respectively.

**Figure 3:**
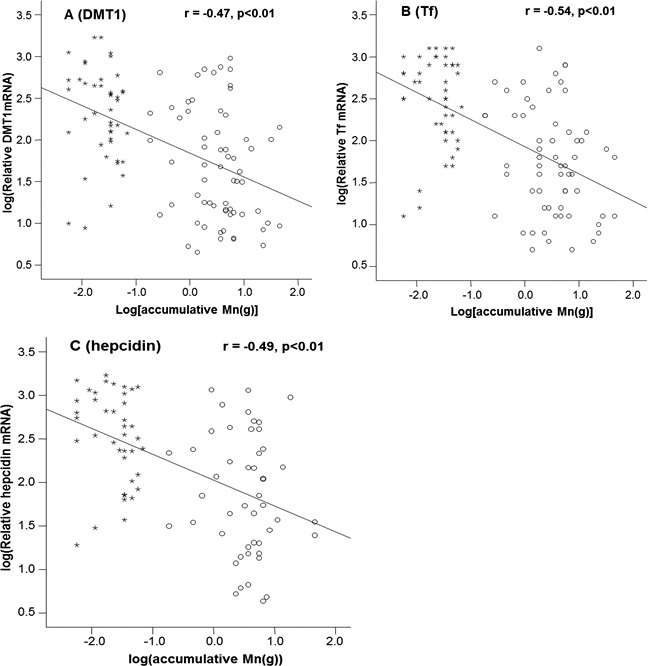
Correlations between DMT1, Tf and Hepcidin and accumulative Mn-exposure. Stars represent the control group and circles represent for the Mn-exposure group. (A) DMT1 expression was inversely correlated with accumulative Mn-exposure (*r* = − 0.47; *p* < 0.01). (B) Tf expression was inversely correlated with accumulative Mn-exposure (*r* = − 0.54; *p* < 0.01). (C) Hepcidin expression was inversely correlated with accumulative Mn-exposure (*r* = − 0.49; *p* < 0.01).

Mn concentrations in erythrocytes were significantly correlated with the cumulative Mn exposure among the study subjects (*r* = 0.46, *p* < 0.01) (Fig. 2A), so were the Mn concentrations in plasma (r=0.59, *p* < 0.01) (Fig. 2B). These positive correlations reflect a job-related Mn exposure. Importantly, the liner regression analysis revealed that the mRNA levels of all 3 Fe transport proteins, i.e., DMT1, Tf and Hepcidin, were significantly decreased as the cumulative Mn exposure increased (*p* < 0.01; Fig. 3), suggesting a Mn exposure-associated alteration in Fe regulatory proteins.

## Discussion

The present study clearly demonstrated that occupational exposure to Mn among smelters is associated with decreased mRNA levels of proteins related to Fe metabolism such as DMT1, Tf, and hepcidin. Our observation provides additional evidence to support the theory that Mn-induced neurotoxicity is due in part to the disturbed Fe metabolism (Cowan et al., 2009a; Cowan et al., 2009b). The data from the current study also suggest that at the airborne Mn concentration near the MAC and TLV (0.2 mg/m^3^), the Mn-caused adverse health effect is evident.

From the chemical point of view, Mn and Fe are situated next to each other in the Period Table. This unique chemical property allows Mn to compete with Fe in absorption, plasma protein binding, and membrane transport (Whittaker, 2003; Roth & Garrick, 2003; Zheng, 2005). The Fe supplement is mainly derived from the absorption in the duodenum, which account for about 10% of the total body need (Knutson et al., 2005; Weiss, 2009). Since DMT1 and Tf are involved in the Fe absorption and transport processes, the decreased expression of both proteins as a result of chronic Mn exposure may partly contribute to a decreased Fe concentration in the systemic circulation.

In our previous animal and cell culture studies, we found that Mn completes with Fe for the 4th binding site in the [Fe-S] cluster of iron regulatory protein-1 (IRP1); the binding allows IRP1 to bind to the stem-loop structures in 3′-untranslated region (UTR) of mRNAs encoding TfR and DMT1 and therefore to stabilize the expression of both proteins. As a result, the expression of TfR and DMT1 increases in the choroid plexus, leading to an increased cellular uptake of Fe and facilitated transport of Fe from the blood to CSF (Li, Zhao & Zheng, 2005; Wang, Li & Zheng, 2006; Zheng, 2005). In the current study, we found a decreased, but not an increased DMT1 mRNA in human blood. Several factors may explain the discrepancy between animal and human studies. First, in our earlier animal studies we did 30-day subchronic Mn exposure (Wang, Li & Zheng, 2006), while the current study was conducted in a human population with long-term, low-level Mn exposure. The different exposure scenario may lead to different DMT1 regulation, which can be tissue-specific and species-specific. Second, the expression of proteins in erythrocytes may not necessarily reflect the expression of the proteins in a specific tissue. Our earlier animal studies reveal the upregulation of DMT1, which occurs in the choroid plexus, a barrier that is responsible for the bi-directional Fe transport between the blood and the CSF, whereas this study determined the DMT1 expression in the blood cells. A reduced expression of DMT1 in blood cells may partly explain a reduced uptake of Fe in the red blood cells as seen in Table 3.

Additionally, the increased Fe concentration in the CSF in our animal studies is the net result of all the Fe-related transporters including DMT1, TfR and MTP1 in the choroid plexus. Based on our previous *in vivo* and *in vitro* data, DMT1 appears to mediate the Fe efflux (from the CSF to blood) transport, whereas TfR mediates the Fe influx (from the blood to CSF) and MTP1 functions to expel Fe from the cells to the CSF, all of which contribute to the increased Fe concentration in the CSF (Zheng & Monnot, 2012). Hepcidin, an endogenous Fe regulatory peptide, has been shown capable of inhibiting MTP1. In the Fe overload status, the hepcidin level increases; an ensuing inhibition of MTP1 activity in the gastrointestinal epithelia leads to a reduced transport of Fe to the blood stream (Adamsky et al., 2004; Gehrke et al., 2003; Theurl et al., 2008). A decreased hepcidin level in the current study may reflect the consequence of the reduced blood level of Fe.

The limitation of the current research pertains to the limited sample size. While many observations are statistically significant, the larger sample size will allow for a better delineation of some borderline correlations such as the relationships between Fe transporter proteins and Fe concentrations in blood. Additionally, the male workers were about 4–5 times more than the female workers; this large difference limits ability of the current study to reveal statistically the gender differences in changes of Fe transporter proteins under the influence of Mn exposure. Future studies shall expand the study population and include more female subjects.

In summary, this study demonstrates that the expressions of genes involved in Fe metabolism, particularly DMT1, Tf and hepcidin, are significantly decreased in the blood of Mn-exposed workers. These findings may be of help in exploring the mechanism by which Mn exposure induces movement disorders in humans. The research toward understanding of Mn–Fe interactions will offer new options for treatment of Mn intoxication, which warrants further exploration.

## Supplemental Information

10.7717/peerj.2413/supp-1 Data S1 Raw Data

## Additional Information and Declarations

### Data Availability

The following information was supplied regarding data availability: The raw data has been supplied as Data S1.
